# COMVC-19: A Program to protect healthcare workers’ mental health during the COVID-19 Pandemic. What we have learned

**DOI:** 10.6061/clinics/2021/e2631

**Published:** 2021-11-10

**Authors:** Pedro Fukuti, Caroline Louise Mesquita Uchôa, Marina Flaborea Mazzoco, Isabella D'Andrea Garcia da Cruz, Mariana V.F. Echegaray, Eduardo de Castro Humes, Júlia Belizário Silveira, Talita Di Santi, Euripedes Constantino Miguel, Felipe Corchs, COMVC-19 program, Daniel Fatori, Guilherme Campello, Gabriel M. de Oliveira, Felipe C. Argolo, Felipe de M. Ferreira, Gustavo Machado, Adriana Argeu, Graça Maria Ramos de Oliveira, Antônio de Pádua Serafim, Luciana de Lima Siqueira, Luciane de Rossi, Izabel Cristina Rios, Talita Rodrigues de Oliveira, Leilane C. K. Antoniazzi, Daniel Augusto Mori Gagliotti, Emílio Abelama, Paulo Novais de Oliveira, Aline Villalobo Correia, Luca Schilling Gonçalves, Liana Silva Tortato, Wagner Machado Moraes Busato, Flávio Guimarães-Fernandes, Marcos Alves, Oswaldo Ferreira Leite, Patrícia de Campos Lindenberg Schoueri, Márcio de Assis Roque, Silvia Stahl Merlin, Giovana Cardoso Machado Boer, Paulo Clemente Sallet, André Malbergier, Mariana Abrahão Spedo, Carla Satie Kamitsuji, Elizabeth de Faria, Moacyr Vergara de Godoy Moreira, Arthur Kaufman, Carmita Abdo, Marco de Tubino Scanavino, Selma Lancman, Hermano Tavares, Guilherme Polanczyk, André R. Brunoni, Orestes V. Forlenza, Tarcísio Eloy Pessoa de Barros-Filho

**Affiliations:** I Hospital das Clinicas HCFMUSP, Faculdade de Medicina, Universidade de Sao Paulo, Sao Paulo, SP, BR.; II Grupo de Assistencia Psicologica ao Aluno, Faculdade de Medicina FMUSP, Universidade de Sao Paulo, Sao Paulo, SP, BR.; III Departamento de Psiquiatria, Faculdade de Medicina FMUSP, Universidade de Sao Paulo, Sao Paulo, SP, BR.; Hospital das Clinicas HCFMUSP, Faculdade de Medicina, Universidade de Sao Paulo, SP, BR

**Keywords:** Mental Health, Healthcare Professionals, Pandemic, Psychological Distress, SARS-CoV-2

## Abstract

**OBJECTIVE:**

In 2020, the COVID-19 pandemic brought a work and stress overload to healthcare workers, increasing their vulnerability to mental health impairments. In response, the authors created the COMVC-19 program. The program offered preventive actions and mental health treatment for the 22,000 workers of The Hospital das Clinicas da Faculdade de Medicina da Universidade de São Paulo (HCFMUSP). This paper aims to describe its implementation and share what we have learned from this experience.

**METHODS:**

Workers were able to easily access the program through a 24/7 hotline. Additionally, a mobile phone app that screened for signs and symptoms of emotional distress and offered psychoeducation and/or referral to treatment was made available. Data from both these sources as well as any subsequent psychiatric evaluations were collected.

**RESULTS:**

The first 20 weeks of our project revealed that most participants were female, and part of the nursing staff working directly with COVID-19 patients. The most frequently reported symptoms were: anxiety, depression and sleep disturbances. The most common diagnoses were Adjustment, Anxiety, and Mood disorders.

**CONCLUSIONS:**

Implementing a mental health program in a multimodal intervention was feasible in a major quaternary public hospital. Our data also suggests that preventive actions should primarily be aimed at anxiety and depression symptoms, with a particular focus on the nursing staff.

## INTRODUCTION

The rapid emergence of the COVID-19 pandemic, in 2020, shook the world. As the number of cases rose and approximately 14% of infected patients presented with severe symptoms and required hospitalization (1) there was a dramatic increase in demand for medical resources burdening the health system capacity of many countries (2). In answer to this challenge, numerous healthcare institutions initiated radical adaptations including: new resource reallocations, the deferment of non-emergency consultations and surgeries, and an increase in hospital bed capacity (2). Brazil was hit particularly hard - as of March 2021, one year after the first case was reported, more than 10 million people have tested positive for the SARS-CoV-2 infection and there have been over 300,000 casualties (3,4).

The Hospital das Clínicas da Faculdade de Medicina da Universidade de São Paulo (HCFMUSP), the teaching hospital of the University of Sao Paulo Medical School (FMUSP) and Brazil's largest public hospital, was designated as the reference medical center for the treatment of severe COVID-19 cases in the city of São Paulo and its surrounding area (approximately 22 million inhabitants). The main building of the hospital, the Central Institute, was entirely revamped – increasing its capacity to 900 hospital beds (400 of them in Intensive Care Units [ICUs]) to assist exclusively with COVID-19 cases. The other HCFMUSP clinical institutes were adapted to receive non-COVID-19 patients to decrease exposure and prevent the spread of disease within the hospital. Most of the workforce (21,000 individuals including medical residents) were reassigned to exert their efforts exclusively either at the Central Institute or in other areas during the pandemic crisis.

Healthcare workers (HCWs) have been particularly affected during the pandemic as they have struggled with work overload, increased risk of contagion, and major changes in their personal routines (5). This has led to an increase in physical exhaustion and psychological distress which are all risk factors for developing mental illness (6,7). In fact, studies have already shown that during the pandemic, HCWs have presented with a higher incidence of psychiatric symptoms and emotional distress compared to the general population (7,8). It is critical, then, that HCWs have ready access to mental health support as a protective factor (6).

Anticipating an upsurge in mental health problems that would overextend the regular mental health resources available for our workers, a multidisciplinary taskforce of mental health specialists from different groups within HCFMUSP were gathered to articulate appropriate actions. These actions aimed to promote psychological well-being and provide mental health support and rehabilitation to our hospital colleagues. We named the program “COMVC-19”^1^: Mental Health and Psychosocial Well-being Personal Protective Equipment for the Health Professionals involved in the struggle against the COVID-19 crisis.” The program was officially implemented on April 3rd, 2020, three weeks after the World Health Organization (WHO) declared the COVID-19 crisis a pandemic. It was designed according to the guidelines of the Inter-Agency Standing Committee (IASC) Guidelines on Mental Health and Psychosocial Support (MHPSS) in Emergency Settings (9) - a framework that emphasizes preventive as well as assistive actions. COMVC-19 has four integrated axes: a. Prevention (Health Promoting Actions); b. Assistance (Mental Health Treatment); c. Education; d. Research (Table 1).

In addition, a smartphone application (app) named COMVC app was developed (10,11). The app allowed users to answer mental health questionnaires and then classified their responses according to symptom severity, while also providing the user with immediate feedback. Mild and moderate cases receive a list of mental health educational videos tailored to their individual needs, while severe cases are referred for treatment. Though specifically designed to support the mental health of our HCWs, the app is also available for download to any individual outside the institution.

The objective of this manuscript is to describe and discuss the characteristics of the program (Table 1) and present some of our data gathered through the hotline, Psychiatric and Psychotherapy assistance, and the COMVC app. As there is a growing risk of subsequent pandemics, we hope that this information will be helpful to other healthcare systems enduring some of the same challenges.

## METHODS

Psychiatry residents were assigned to provide assistance to our HCWs and manage the hotline. In order to train them for this new situation a 5-week online course was created including the following topics: psychiatry in humanitarian crises, psychiatric interviews, mental health and risk assessment screening via the hotline, telepsychiatry, adjustment disorders, anxiety disorders, stress disorders, mood disorders, brief psychotherapy techniques, mindfulness, and relaxation techniques. All residents also took the Johns Hopkins University’s Psychological First Aid (PFA) course (12). This course was chosen due to its simple and straightforward structure and the existence of free online availability with Brazilian Portuguese subtitles. Both are available to the public (Table 1).

### Hotline

The hotline phone number was advertised extensively to the hospital staff through institutional e-mail, social media posts, and local banners as a 24/7 service offering mental health support and an entry point to psychiatric and psychotherapeutic treatment. The phones were overseen by a second-year psychiatry resident who was trained as described above. When a call was received, the caller was provided with immediate psychological support including respectful and qualified listening, and then screened for mental health issues, with referrals to appropriate treatment according to their needs.

Although information about the hotline was extensively advertised to the hospital staff, we soon became aware that some workers did not feel comfortable with the idea of asking for emotional support. Hence, in parallel, we started an active search strategy, specifically identifying those who appeared to be experiencing mental health symptoms or psychological distress to encourage them to seek help. This process was conducted by members of our team working directly in the Central Institute but every worker who received our PFA training could approach more acute cases.

The principles of PFA were used on the hotline, classifying the caller by their level of distress and disability. Individuals who were “doing well” and were able to handle their daily demands were assigned to the “Eustress group,” as they benefited from the support over the phone and were informed that they should call back if they needed. Workers who presented with mild symptoms and who might have had coping resources were assigned to the “Distress group.” The need for referral in these cases depended on clinical and risk factors, such as contact with death, low social support, previous mental illness, and risk-taking behavior. Finally, workers who showed more significant impairments, especially involving their agency for self-care, were assigned to the “Dysfunctional group” and referred to psychiatric consultation, brief supportive psychotherapy, or, in more urgent cases, to psychiatric emergencies services (see Table 1 for link to training videos for all these interventions).

After each call, residents would fill out a Google Form containing the patient’s sociodemographic information such as sex, age, hospital function (administrative, nursing, medical or other), sector of the hospital where they were working (COVID-19 or non-COVID-19), their main psychiatric issue, and finally the need for referral to a psychiatric consultation, brief supportive psychotherapy, or emergency unit.

### Web-based Clinical consultations and brief supportive psychotherapy sessions

Psychiatric consultations and follow-ups were provided to individuals referred from the hotline (or other means) and consultations were scheduled within 24 hours in most cases. Psychiatry residents delivered 40-60-minute consultations under the supervision of experienced psychiatrists. These consultations involved diagnosis. choice and prescription of appropriate medication, as well as education regarding mental health, sleep hygiene, and/or anxiety management. Subsequent visits, when necessary, were scheduled for a date deemed appropriate.

Supportive Psychotherapy sessions also lasted 40-60 minutes and were delivered via online settings, mainly by residents. Initially, individuals were offered 4 sessions, but this could be extended if necessary. Residents were supervised by experienced therapists of diverse theoretical affiliations (e.g., psychodynamic, cognitive behavioral therapy, etc.) from our psychotherapy unit.

After the consultation, residents were asked to fill out a Google Form containing information such as previous psychiatric or psychotherapeutic treatment, main symptoms, the need for introducing or augmenting psychotropic medications, the need for medical leave, and finally, their diagnosis based on the International Code of Disease (ICD10) criteria. Residents were also asked to rate the severity of their symptoms based on the Clinical Global Impression Severity scale (CGI-S), a widely and easy to use instrument to measure severity of disease in psychiatry (13).

### COMVC Smartphone Application

The app was advertised extensively and freely offered to all HCWs. Depression and anxiety symptoms were measured using the Patient-Reported Outcomes Measurement Information System (PROMIS) (14), sleep problems were measured using the Single-Item Sleep Quality Scale (15), and burnout symptoms were measured using the Single Item Measure of Emotional Exhaustion (16). All instruments were selected because of their brevity and robust psychometric properties. Based on validated criteria, an algorithm was used to classify responses in real-time into three categories related to severity: No symptoms, Moderate, and Severe. This was done for each of the four domains according to the following criteria: Depression (11-16 = moderate depression, 17-20 = severe depression), Anxiety (11-15 = moderate anxiety, 16-20 = severe anxiety), Sleep problems (4-6 = moderate sleep problems, 0-3 = severe sleep problems), Burnout (1 = moderate burnout, 2 = severe burnout).

The institutional ethics committee was consulted and approved this study, as part of ongoing research in our hospital concerning the mental health of HCWs (CAAE 30710620.2.0000.0068).

## RESULTS

### The first five months of experience (April 3^rd^ - August 21^st^, 2021)

We report here an overview from the first 20 weeks of hotline operation and admissions (*N*=395), the first consultation in psychiatry and/or psychotherapy admissions (*N*=131), and our app data (downloads *N*=913 and active users *n*=704).

### Hotline for specialized assistance (N=395)

The COMVC-19 program started operating on April 3^rd^, 2020, and after 20 weeks of operation, 395 HCWs were admitted to the program. Among those, 357 (90.4%) of individuals had called the hotline by their own initiative, 32 (8%) individuals were referred by active search and 6 (1.5%) were referred by the emergency department.

As described in Table 2, among the HCWs, most were female and from the nursing staff. It is noteworthy that of the 90 physicians seen in our service, 71 (78.9%) of them were medical residents. As expected, most were currently working in the Central Institute in direct assistance of COVID-19 patients.

Interestingly, half of the HCWs reported no previous psychiatric background. Among those who reported a previous psychiatric history, most had depressive or anxiety disorders (Table 3).

Of the 395 HCWs, 182 (46%) were referred exclusively to psychiatric care, 101 (25.5%) to brief supportive psychotherapy, and 89 (22.5%) to both. The remaining 23 (5.8%) had no need for specialized referral.

### Web-based Clinical consultations and brief supportive psychotherapy sessions (N=131)

After the first evaluation on our Hotline (*N*=395) and referral to consultation (*n*=372), a full dataset from 131 individuals (35.2%) was collected (Mean age=36.5, S.D. 12.5). Missing data were due to individuals who did not fill out forms after each psychiatric consultation. The most frequent symptoms reported were anxiety-related, depressive mood, and sleep disturbances (Table 4).

The most prevalent diagnoses were Adjustment Disorder, Unipolar Depressive episode, Anxiety Disorders, and Mixed Anxiety and Depressive Disorder (Table 5).

The majority (25 of the 38 [65.7%]) of the participants with stress-related diagnoses (Adjustment disorder, Acute Stress Reaction, and Post-traumatic Stress Disorder), had no history of previous mental health disorder, thus indicating that it was directly related to their work during the COVID-19 pandemic (Table 5).

We were able to measure the severity of symptoms using the CGI-S scale in 114 participants. In 31 (27.2%) of these cases, symptoms were considered mild, while in 54 (47.4%) of cases, symptoms were considered moderate.

Medical leave due to psychiatric symptoms was indicated for 13 of 131 (9.9%) participants.

### The COMVC app

The COMVC app was launched on June 18, 2020. By August 18, 913 HCFMUSP workers had downloaded the app. Among those, 704 were active users who reported mental health symptoms at least once. Among these users, 583 were female (82.8%). We calculated prevalence rates of anxiety (37.4%), depression (23.6%), sleep problems (30.4%), and burnout (75.8%) by estimating users who reported symptoms above the cutoff for severe problems at least once across all available assessment time points (Table 6). The majority (83.1%) of the HCFMUSP workers using the app received at least one referral to the COMVC-19 hotline.

## DISCUSSION

We will first discuss the broad spectrum of the preventive actions that our program comprised and then discuss the results we have presented from our Hotline and COMVC app.

**Figure 1:**
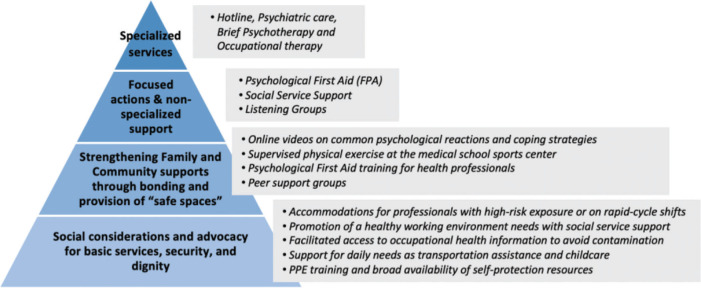
The Inter-Agency Standing Committee (IASC) pyramid.

The IASC Guidelines on Mental Health and Psychosocial Support (MHPSS) in Emergency Settings recommends multiple levels of interventions targeting a spectrum of mental health and psychosocial needs, ranging from essential services to specialized assistance (9,17). The IASC pyramid (Figure 1) summarizes our approach. Note that the base of the pyramid defines initial general support available to a greater number of individuals. Services at this level are preventative in nature, thereby aimed at protecting against greater mental stress. With each incremental step, the intervention strategies become more complex, assistive, and specialized, ideally being needed by a smaller number of individuals.

In situations of stress, making basic environmental changes that target the needs of those affected can be an efficient means of preventing mental health issues (see IASC pyramid base, Figure 1). Indeed, many hospitals throughout the world have prioritized prevention by addressing the basic needs of their workforce: food, rest, housing, less exhaustive work shifts, and providing relaxation and leisure activities (18,19).

Listening. A viewpoint published in *JAMA* summarizes the most common requests made by frontline healthcare workers to their organization during the COVID-19 pandemic. They include the following: “hear me”, “protect me”, “prepare me, “support me” and “care for me” (5). Indeed, feelings of not being listened to by the institution are associated with a higher risk for mental health concerns (6). To address this issue, we monitored work related claims and sources of anxiety via our talk groups, PFA, hotline and assistive activities. We then communicated these concerns to our institutional leadership in an attempt to ameliorate any stressful work conditions in the hospital.

The most common concerns endorsed by hospital staff were 1) a lack of personal protective equipment (PPE), 2) being infected, and 3) infecting their loved ones. Other common concerns were the stress of adapting to new routines, a lack of institutional communication, and work overload. These sources of stress are common among HCWs and have been observed in other services (6,18,19).

Medical residents were particularly concerned about missing practical learning time related to their speciality of choice. Remember, many of these residents were assigned exclusively to treat COVID-19 patients whereas others remained engaged in their original activities. The rotation of medical personnel between COVID-19 and non-COVID-19 sectors was forbidden in an attempt to prevent viral spread. Feelings of injustice and, in turn, contempt towards those colleagues who were not reassigned to COVID-19 sectors, were commonly reported.

The HCFMUSP administrators attempted to improve work conditions by expanding the workforce, establishing 12/36h shifts (12 hours of work, followed by 36 hours of rest), and keeping a low patient/HCW ratio to prevent work overload. The administration also established clearer communication with hospital staff by centralizing COVID-19 related information on our institutional website, including improved protocols for the adequate management of PPE and treatment of COVID-19. HCWs were continually updated on the adequate amount of PPE in the hospital. Indeed, we did not face depletion of any necessary medical supplies. After some time, it was documented that there was a low viral transmission rate within the hospital, allowing for the rotation of medical residents to be reinstated, which alleviated much stress for many HCWs.

It is worth noting that the existence of mental health issues among HCWs has often been ignored by health institutions (20). During this pandemic, this was not the case at our hospital. Mental health care was given the institutional importance that it deserved, hospital staff remained sensitive to mental health issues of their coworkers, and the COMVC-19 program was welcomed by leaders of all sectors. Indeed, many participants called the hotline on the advice of a coworker. Perhaps one positive outcome of the current pandemic was the diminishment of mental health stigma that has been all too common for many years.

Psychoeducation is an intervention that consists of offering mental health information to help individuals to self-care – that is, prevent or cope with mental health issues in order to promote resilience. Web-based videos have been shown to be quite effective in achieving this goal (21). Psychoeducational videos about common psychological problems, coping skills, as well as mindfulness and relaxation techniques to augment psychological resilience, were made available on the institutional website and were available to all (Table 1). Psychoeducation such as this has been highly recommended during the pandemic and has been adopted by similar programs (5,6,18,19).

Psychological First Aid (PFA) is an approach recommended by the World Health Organization (10) to offer initial psychological care to people in a highly stressful situation. One advantage of PFA is that it can be made readily available, as it can be delivered by non-mental health specialists. PFA aims to mitigate acute suffering and assess the need for continued mental health care through a compassionate and supportive presence. Such a presence promotes calmness, guidance, security, and instrumental support adapted to the needs of emotionally overworked people (28). Note that training our staff to act as PFA agents is a second-level action within the IASC pyramid, whereas the delivery of the program itself is part of the third level of the pyramid. In order to gain capillarity throughout the hospital, the COMVC-19 program trained a team of 117 HCWs from various units (e.g., ICU, emergency department, infectious disease and internal medicine) to act as PFA internally in their units in support of their team

Fostering Nurses Group. Most of our participants were female nurses, a population known to be at a higher risk for suffering from mental health issues (6,8). The Fostering Nurses Group focused on this population (as part of the third level of the pyramid, Figure 1). The program was based on evidence that decompression actions and psychological approaches related to the nursing work experience can prevent burnout syndrome (22). Fear of contaminating their families was the most prevalent topic brought up at these meetings. Worrying more about contaminating others than themselves was also observed in non-HCWs (23). Other prevalent topics were the stress related with having to deal with the large number of new hires (as they were not familiar with the daily hospital routine) and miscommunication between the teams.

Physical exercise is well known to augment resilience and mitigate mental health issues. Recent research has shown that physical activity is the preferred coping behavior of 56% of HCWs (24). Unfortunately, many of our HCWs did not have a place to exercise because the lockdown hampered their access to open areas, fitness centers, or other places where physical activities would usually take place. Indeed, the Sports Center at our institution, administered by the medical students, had been idle since the beginning of the pandemic. In response, the students, supported by the institution, recently re-opened the center exclusively for frontline workers and 533 of them participated in professionally-guided physical activity that included physical conditioning, Pilates and jogging.

### Hotline for specialized assistance

Hotlines for mental health support and easy access to mental health care have been highly recommended and are used in many mental health services in the world (24). (Note that hotlines represent the access to the top part of the pyramid, Figure 1). Among the 395 HCWs who sought assistance, 308 were female (79.5%), and 123 were on the nursing staff (31.1%). 113 (28.6%) of our participants were female nurses. Some nurses reported that they had never experienced so much fear of going to work. Due to a lack of hospital beds, some nurses reported their concern that they would not be able to receive treatment if they contracted the disease. Some reported that they feared for their own lives. Worries about colleagues who were sick were also common. Indeed, having someone close to you that has contracted the virus has been associated with a higher risk of having psychiatric symptoms (6).

After nurses, physicians were the next most likely group to seek help and exhibit psychiatric symptoms (n=90; 22.7%). Importantly, the presence of such symptoms in physicians is associated with a higher risk of making medical errors (25). Among these physicians, 71 (78.9%) were medical residents. It has been advocated that younger doctors should be the main force on the frontlines, as they are less likely to develop severe forms of COVID-19 (1). Though this may be true, our findings should be a warning that, at the same time, this group is at greater risk for suffering mental health issues (6). Some residents reported being so frustrated with their assignment to the frontline that they seriously considered quitting their residency program. Any resident who shared such a sentiment with the Residency Commission of our teaching hospital (Comitê de Residência Médica), was referred to our unit for psychological assistance. In the majority of cases, these individuals chose to remain in the profession after a brief leave and/or upon receiving psychological support. It is worth noting that numerous residents across many specialties actively volunteered to work on the frontline (including 11 psychiatry residents). Perhaps volunteering during a crisis serves as a protective mental health factor for a subset of these individuals.

The implementation of an active search strategy, turned out to be very effective in amplifying the scope of our actions (32 individuals reported that they would not have sought help otherwise received support through this approach). It is noteworthy that 11 of these individuals were physicians (34.4%). Previous studies have shown that it is common among physicians to not recognize their own need for treatment and therefore not seek help (20,25,26). Perhaps surprisingly, then, many of our physicians welcomed our approach, reporting that they felt the need to talk and decompress, but would not take the first step by themselves. These findings suggest that an active approach might be helpful in this situation.

The number of referrals to our psychotherapeutic (101; 25.5%), psychiatric (182; 46%) or both (89; 22.5%) treatment services shows that both modalities of treatment have been of great utility during the current pandemic. Indeed, many other similar programs offer both treatment services (18,19).

### Web-based clinical psychiatric consultations and brief supportive psychotherapy

Web-based clinical psychiatric consultations and brief supportive psychotherapy have been recommended by the WHO with cumulative evidence showing that these interventions have had therapeutic value during the current pandemic (27).

Symptoms of anxiety, depression and sleep disturbance were most commonly reported by our participants, consistent with epidemiologic surveys during this and previous pandemics in other countries (5,6,23).

The most prevalent diagnosis in the current sample was Adjustment Disorder, followed by anxiety and depressive disorders. Half of the HCWs within the HCFMUSP had no previous psychiatric history. Where a past diagnosis did exist the most common was also depressive or anxiety disorders, or both. These findings suggest that the current pandemic has not only produced a surge in new diagnoses, but has also induced the recurrence of previous disorders. Fortunately, in the present group, severity of illness was mostly moderate or mild. Indeed, medical leave was needed in only 13 of 131 (9.9%) cases.

### The COMVC app

The COMVC app was accessed by numerous HCWs in the hospital who downloaded and responded to the scales therein. In a relatively short period of time (2 months), the app was downloaded 913 times. Thus, these app data may represent an estimation of the prevalence of pandemic-related psychiatric symptoms among the HCWs of HCFMUSP. Examination of the responses to the PROMIS and Sleep Quality Scale revealed a high prevalence of anxiety, sleep problems and depression among our workers. The app has proven to be a useful tool to privately and conveniently access a greater number of our workers, screen them for mental health issues, and offer psychoeducation or referral to treatment.

### Limitations

Due to the multimodal nature of the COMVC-19 Program and the pressure for its fast implementation, interventions could not always be systematically administered, measured or controlled with comparison groups. For example, our data do not include those who did not seek help or search for other services. The problem of missing data due to participants who did not follow through with filling out all evaluation forms further limited our efforts. In addition, though the program was not specifically designed for research, the current presentation offers preliminary data that can be used to devise controlled studies of these phenomena. Our goal here was to offer the field details of our initial efforts and preliminary data reflecting the impact of this pandemic on our HCWs.

## CONCLUSIONS

The COMVC-19 program represents a multimodal intervention, created in response to the COVID-19 pandemic. Our program is based on previous studies, the experiences of other countries, and on the IASC recommendations for facing disaster situations. Our goal is to mitigate mental health issues in the HCWs of Brazil’s largest public hospital. To this end, COMVC-19 is focused on preventative and assistive actions.

Although myriad mechanisms are involved in the emergence of mental health disorders (28), psychiatrists do not regularly engage in proactively modifying environmental risk factors to prevent mental illness. The COMVC-19 program comprised broad preventative actions such as listening to the HCWs, implementing improvements in work conditions, psychoeducation, broad training in Psychological First Aid and facilitating the practice of exercise and sports for all frontline workers. We also fostered a nurses talk group led by psychotherapists. Since nurses, particularly those on the COVID-19 frontlines, were most likely to engage with our program, these individuals should be the focus of future preventive strategies. The present intervention could serve as a proof of concept for future randomized control trials in this regard.

From the assistive actions, the hotline, and our outpatient service designed specifically to assist frontline HCWs, we have noted many similarities with the mental health issues observed in the general population. For instance, women are more sensitive to their mental health status, more frequently seek help, and have reported more psychiatric symptoms during the pandemic (28,29). Accordingly, female HCWs made up the majority of those who sought assistance in our program (∼78%) and accessed the app (∼83%). Similarly, during the pandemic, anxiety and depression symptoms have been the most prevalent psychiatric symptoms observed in the general population (28% and 24%, respectively) (23,29) and the most prevalent among our HCWs (though the rates are much higher for HCWs as is expected [72.5% and 45%, respectively]).

Our experience also suggests that web-based mental health psychiatric consultation and brief supportive psychotherapy are effective. Likewise, apps such as the COMVC app are useful for screening, referral, and to offer preventative information (such as videos) that can help HCWs cope. We have shown that the initial version of our app is viable, and that this service should be scaled up for larger populations.

Our objective was to share with other health institutions and mental health specialists what we have observed and learned after 5 months of implementation of our COMVC-19 program. We hope that the insights shared here have offered some valuable insights to other institutions interested in implementing similar services. It is noteworthy to explicitly state that though this is a period of great suffering and concern, it is also a time where we might seize great opportunity. At our institution, we were impressed by how available our HCWs made themselves, contributing in one way or another to help those in need. Any existing difficulties among our staff, of a personal or ideological nature, were set aside while our institution banded together around a common goal. A feeling of institutional cohesion was created that had not been observed for a long time. This cohesion greatly facilitated the implementation of all modalities within the program. Even the need for financial resources was overcome with generous support from many different sectors of society that donated resources for the creation of a teleconsultation room and the COMVC app.

Approximately one month after the onset of the program, an email was sent asking participants to evaluate the service provided by the COMVC-19 Program using a 0 to 10 rating scale. We received feedback from 70 respondents. Of those respondents, 88.6% assigned a grade of 8 or higher and affirmed that they would recommend the program to others. Positive comments such as “I was welcomed in the moment I needed the most! were observed frequently. We are confident that the positive comments that we have received from our HCWs participants would mirror what their patients would say about them. Afterall, COMVC-19 was devised by HCWs, and represents HCWs helping HCWs. Such solidarity and proactivity towards a common goal during this dreadful pandemic is the greatest legacy of this experience and should inspire our paths after the pandemic.

### COMVC-19 program collaborators

**Daniel Fatori**, **Guilherme Campello**, **Gabriel M. de Oliveira,** **Felipe C. Argolo**, **Felipe de M. Ferreira**, **Gustavo Machado, Adriana Argeu** (COMVC app). **Graça Maria Ramos de Oliveira**, **Antônio de Pádua Serafim**, **Luciana de Lima Siqueira**, **Luciane de Rossi**, (Psychological First Aid). **Izabel Cristina Rios**, **Talita Rodrigues de Oliveira**, **Leilane C. K. Antoniazzi**, (Fostering Nurses and humanization group). **Daniel Augusto Mori Gagliotti**, **Emílio Abelama Neto**, **Paulo Novais de Oliveira Junior**, **Aline Villalobo Correia**, **Luca Schilling Gonçalves**, **Liana Silva Tortato**, **Wagner Machado Moraes Busato**, **Flávio Guimarães-Fernandes**, **Marcos Alves**, (Psychiatric Care). **Oswaldo Ferreira Leite Netto**, **Patrícia de Campos Lindenberg Schoueri**, **Márcio de Assis Roque**, (Psychotherapy Sector). **Silvia Stahl Merlin**, (Active Search) **Giovana Cardoso Machado Boer** (IT). **Paulo Clemente Sallet**, **AndréMalbergier**, **Mariana Abrahão Spedo**, **Carla Satie Kamitsuji**, **Elizabeth de Faria**, **Moacyr Vergara de Godoy Moreira**, **Arthur Kaufman**, **Carmita Abdo**, **Marco de Tubino Scanavino**, **Selma Lancman**, **Hermano Tavares**, **Guilherme Polanczyk**, **André R. Brunoni**, **Orestes V. Forlenza**, **Tarcísio Eloy Pessoa de Barros-Filho** (program conception and institutional support). All of the members are from Hospital das Clinicas HCFMUSP, Faculdade de Medicina, Universidade de Sao Paulo, SP, BR.

## AUTHOR CONTRIBUTIONS

Fukuti P, Uchôa CLM, Mazzoco MF, Cruz IDAG, Echegaray MVF, Humes EC, Silveira JB, and Di Santi T were responsible for the manuscript draft, analysis and interpretation of data. Humes EC, Corchs F and Miguel EC were responsible for revising the manuscript critically. All authors and collaborators were responsible for conception and design of the program.

## Figures and Tables

**Table 1:** The components of the COMVC-19 program.

Action and Description	Subjects (n)	Details
**Preventive (Health Promoting Actions)**		
**Listening to the Employees** HCWs provided feedback via talk-groups and hotline evaluation regarding what could be done to improve their working conditions. The requests were passed along to the institutional leadership.	Participants of the Fostering Nurses Group, rPsychological First Aid (PFA) groups, and every employee who accessed the hotline	Any reports of stressful working conditions as well as other requests of the employees that could potentially improve their psychological wellbeing were transmitted to the institutional leadership
**Psychological First Aid (PFA)** Training in psychological first aid techniques was given to non-specialist hospital staff leaders.	117 HCWs were trained throughout the hospital	This consisted of humane, supportive and practical help to fellow human beings suffering serious crisis events. The course is available at https://www.coursera.org/learn/psychological-first-aid
**Psychoeducational videos** We developed a database of psychoeducational videos (5 minutes or less) related to mental health and self-care during the pandemic delivered by mental health specialists of our Hospital.	15 videos	The videos were made available on an institutional website divided into three themes: General information about COVID-19, How health workers can protect their mental health, How to deal with emotionally stressful events and mental disorders. https://sites.google.com/hc.fm.usp.br/comvc-19/v%C3%ADdeos-colaboradores-do-hc?authuser=0#h.xoem956gjpo9
**Motivational Videos** Videos with positive messages a) emphasizing the relevance of their efforts and b) showing appreciation for the health care workers on the frontline	4 videos and a grand round dedicated to the frontline workers	1. https://www.youtube.com/watch?v=v93WcpakAE8&feature=youtu.be 2. https://www.youtube.com/watch?v=SZNpThAhQ2s&feature=youtu.be 3. https://drive.google.com/file/d/1oKyasr6HtwVJpwrUkdGVFk-J5nIBFk2t/view 4. https://drive.google.com/file/d/1HbDiCMdcrIlN5PDeRjJnzkP9BPI5dfOP/view 5. https://www.youtube.com/watch?v=wJjBhUwMSok
**Fostering Nurses Group** A ‘Fostering Nurses’ (‘Enfermagem que Acolhe’) implemented by the Humanization Group (Núcleo Técnico de Humanização, NTH).), with the aim of discussing routines, developing emotional support, and stimulating reflections among colleagues.	246 participants 79 encounters over the first three months	It consisted of talk groups with ∼10 participants and was conducted weekly by psychiatry residents, psychiatrists, and psychotherapists. These groups were offered to the ICU’s, ER’s, and ward’s technical and registered nurses. The hope was for our HCWs to ultimately understand the subjective meanings of their professions.
**Professionally-guided physical activity** Professionally-guided physical activity to the frontline COVID-19 HCWs	533 participants	230 classes over the first month including physical conditioning, Pilates, and jogging
**Assistance (Mental Health Treatment)**		
**Hotline** The Remote Mental Health Support via Hotline available 24 hours a day to HCWs who may need mental health support. It is the primary entry access to all available therapeutic actions.	21 second-year residents on 24/7 shifts. 357 calls	The hotline offers a qualified initial screening followed by referral to psychiatric consultation and/or brief supportive psychotherapy when necessary. A training video on how to manage a hotline and screen for mental health issues is available. https://www.youtube.com/watch?v=YgxMGo6wUOQ&feature=youtu.be
**Psychiatric Assistance** Provided web-based psychiatric assistance by psychiatric residents under experienced supervision.	30 1st, 2nd and 3rd year residents + 15 medical supervisors	A training video on about web-based psychiatric consultation used to train our residents was made available: https://www.youtube.com/watch?v=gQKOBoOzT38
**Brief supportive psychotherapy** Brief supportive psychotherapy sessions by psychiatric residents under experienced supervision on various theoretical frameworks, both psychodynamic and cognitive-behavioral orientations.	More than 100 participants, 400 sessions 40 psychiatry residents + 20 volunteers psychotherapists	Video explaining principles of brief psychotherapy https://www.youtube.com/watch?v=aHeW3_Yfzzk&feature=youtu.be
**Active search/screening** Psychiatrists working on the Central Institute (COVID-19) or peers trained in PFA would approach HCWs who seemed to be in distress and would kindly suggest that they call the hotline	38 individuals	The leadership committee and leaders in key hospital areas (Intensive Care Units and Emergency Settings) were particularly targeted.
**Rehabilitation Program** During the early phases of the program, we noticed that many workers who contacted us were on medical leave, either for mental health issues or general medical reasons. Thus, we included in the program an initiative that would promote and strengthen their employment relationship, develop and adapt their abilities to return to work, and follow them up after their return to work during such a stressful time.	10 HCWs assisted	The Occupational Therapy Department, through its staff and residents, started leading a branch of the program responsible for offering these services also via video calls. Video on occupational therapy https://www.youtube.com/watch?v=Ya4yvL-Xkds&feature=youtu.be
**COMVC APP Smartphone Application.** Smartphone application (app), named COMVC, for healthcare workers and the general public aimed at disseminating brief educational videos on the COVID-19 pandemic and mental health, monitoring symptoms and providing customized feedback to individual needs.	913 HCFMUSP workers installed the app. 704 were active users who reported mental health symptoms at least once.	“COMVC APP, iOS Version.” Apple App Store. 2020. apple.co/2UX32rY. “COMVC APP, Android Version.” Google Play Store. 2020. bit.ly/37Ie6P4.
**Educational**		
**Videos to train professionals** Psychoeducational videos related to mental health care and the type of assistance offered by the program	10 videos	Available at https://sites.google.com/hc.fm.usp.br/comvc-19/v%C3%ADdeos-colaboradores-do-hc?authuser=0#h.v6vd06l22v53
**Scientific meetings** A YouTube channel with scientific meetings focused on COVID-19 and its impact on mental health	9422 people have been assessed until the submission of the paper	https://www.youtube.com/channel/UCHgcdM65SzU1-6L24MyDMcA
**Research**		
**Screening of hospital HCWs mental health status**	Sent to all the 21,000 HCWs	An online survey composed of self-report questionnaires and psychometric instruments assessing the most important psychological reactions seen in this kind of situation.
**Investigating HCWs emotional reactions to the pandemic**	40 participants.	Application of semi-structured interviews to a few selected participants who worked on the front line to qualitatively explore their emotional reactions to the crisis
**Screening the mental health of young during the pandemic**	Around 7000 answers	An investigation of the impact of the crisis on young children and adolescents from 5-17 years old throughout Brazil.
**Impact of SARS-CoV-2 pandemic on mental health in the elderly**	500 participants	Prospective assessment of the mental state and characterisation of the emotional distress among older participantsfrom the psychogeriatric clinics at HCFMUSP
**Neuropsychiatric Manifestations of SARS-CoV-2: a cohort study**	1200 participants	Investigated the direct impact of the SARS-CoV-2 in the Central Nervous System (including its psychiatric and neurologic manifestations).

**Table 2:** Demographics of workers admitted in the COMVC-19 mental health assistance program.

General Information	Number of workers (%)
Admission in the program	395 (100%)
Hotline	357 (90.4%)
Active search	32 (8.1%)
Emergency Department	6 (1.5%)
Sex	
Female	308 (77.9%)
Professional position	
Nursing staff	123 (31.1%)
Physicians	90 (22.7%)
Administrative	92 (23.2%)
Physiotherapists	9 (2.3%)
Laboratory technicians	7 (1.8%)
Other	75 (18.9%)
Sector	
Central Institute (COVID-19 patients only)	166 (41.9%)
Heart Institute	49 (12.4%)
Cancer Institute	37 (9.3%)
Outpatients Institute	31 (7.1%)
Others	112 (28.3%)

**Table 3:** Psychiatric status of workers admitted in the COMVC-19 mental health assistance program.

Previous psychiatric history	N (%) Total = 395 (100%)
None	181 (45.7%)
Depressive Episode/Symptoms	78 (19.7%)
Anxiety disorders	53 (13.4%)
Mixed Anxiety-Depressive Disorder/Symptoms	16 (4%)
Panic Attacks/Disorder	10 (2.5%)
Others	57 (14.4%)

**Table 4:** Prevalence rates of psychiatric symptoms in first psychiatric consultations or psychotherapy sessions.

Most Common Symptoms	N (%) Total = 131 (100%)
Anxiety	95 (72.5%)
Depressed Mood	59 (45%)
Sleep Disturbances	54 (41.2%)
Irritability	26 (19.8%)
Panic Attacks	15 (11.4%)
Fatigue	12 (9.2%)

**Table 5:** Main diagnosis as of the first consultation in the COMVC Program (psychiatry and psychotherapy).

Main diagnosis	N (%) Total = 131 (100%)
Adjustment Disorder	31 (23.7%)
Unipolar depressive episode	29 (22.1%)
Anxiety Disorders	24 (18.3%)
Acute Stress Reaction	6 (4.6%)
Mixed Anxiety and Depressive Disorder	11 (8.4%)
Burnout	5 (3.8%)
Panic Disorder	4 (3.1%)
Insomnia	4 (3.1%)
Bipolar Disorder	3 (2.3%)
Post-Traumatic Stress Disorder	1 (0.8%)
No diagnosis	3 (2.3%)
Other diagnosis	10 (7.6%)

**Table 6:** Prevalence rates of psychiatric symptoms based on assessment via the COMVC app.

Diagnoses COMVC app	N (%) Total = 704
Severe burnout	534 (75.8%)
Severe anxiety	263 (37.4%)
Severe sleep problems	214 (30.4%)
Severe depression	166 (23.6%)

## Footnotes

1 The acronym “COMVC-19” comes from a wordplay between COVID and COMVC, a short version for *“com você”* (“with you” or “by your side” in Brazilian Portuguese).
